# The determinants of postpartum contraceptive use in Nigeria

**DOI:** 10.3389/fgwh.2023.1284614

**Published:** 2023-12-12

**Authors:** Obinna Princewill Anyatonwu, Kelechi Amy Nwoku, Håkan Jonsson, Fredinah Namatovu

**Affiliations:** ^1^ Department of Epidemiology and Global Health, Umeå University, Umeå, Sweden; ^2^ Centre for Demographic and Aging Research at Umeå University (CEDAR), Umeå University, Umeå, Sweden

**Keywords:** family planning, birth spacing, postpartum, women’s health, HBM, fertility

## Abstract

**Introduction:**

Postpartum contraception is vital for maternal and child health, and reduces the risk of infant mortality. The Health Belief Model (HBM) is a widely accepted framework for exploring health behaviors, such as contraceptive use. Therefore, this study aimed to investigate the factors influencing postpartum contraceptive use in Nigeria and to contextualize the findings within the framework of the HBM.

**Methods:**

This study was a secondary analysis of cross-sectional data collected from the Demographic Health Survey conducted in Nigeria (NDHS). In total, 28,041 women were included in this study. Self-reported contraceptive use was the outcome, while the explanatory variables included maternal age, place of residence, region of residence, religion, marital status, educational level, household wealth quintiles, knowledge of the ovulatory cycle, decision-maker for health care, and distance to health care facilities. Descriptive statistics and multivariate logistic regression were used to summarize and identify factors influencing postpartum contraceptive use. The HBM was used to discuss the main findings.

**Results:**

The prevalence of postpartum contraceptive use in Nigeria is 27%. Our findings showed that the odds of using contraceptives during the postpartum period were higher among women who knew their ovulation cycles, lived in urban areas in the southern region, had no distance barriers to health care, and were 25–49 years old. Education, wealth, and marital status also increase the odds of contraceptive use. However, women who lived in the northeast and northwest regions or shared decision-making with their partners had lower odds.

**Conclusion:**

This study highlights the need for region-specific and age-focused interventions to increase contraceptive use in Nigeria. Additionally, increasing accessibility and affordability of contraceptives for younger and economically disadvantaged women, along with promoting women's autonomy in decision-making, can further enhance contraceptive use across Nigeria.

## Introduction

Postpartum contraception, which commences within a year of delivery, is crucial for maternal and child health. It not only mitigates the risks associated with short birth intervals, such as infant mortality but also prevents health risks from unintended pregnancies, including delayed prenatal care and low birth weight (1). According to the United Nations (2), wide-scale adoption of such methods could prevent over a million neonatal and infant deaths and 118,000 maternal deaths globally. The unmet need for postpartum contraception is high in sub-Saharan African countries, where over 30 percent of pregnancies are unintended (3). In Nigeria, family planning and contraceptive use remain markedly low (estimated at 17%) compared to other sub-Saharan countries. For instance, Lesotho (59%), Namibia (56%) and other counties in the region have reported significant higher contraceptive prevalent rates (4, 5). These findings highlight the substantial disparity in contraceptive use between Nigeria and these neighboring countries, indicating the need for improved access to and awareness of contraceptives in Nigeria to enhance family planning and reproductive health.

The Health Belief Model (HBM) is a widely used theoretical framework for understanding health behaviors and designing interventions to promote them. The HBM posits that individuals' decisions to adopt particular health behaviors are influenced by their perceived susceptibility to a health problem, perceived severity of the problem, perceived benefits of the behavior, perceived barriers to the behavior, cues to action, and self-efficacy (6). The HBM provides a valuable lens for exploring how individual beliefs, perceptions, and sociocultural factors shape contraceptive behavior. By examining HBM constructs in the context of postpartum contraception use, we can gain insights into the factors influencing contraceptive utilization and inform targeted interventions.

Perceived susceptibility refers to an individual's perception of the likelihood of experiencing an unwanted pregnancy. Britton et al. (7) found that addressing fertility misperceptions among women with low perceived susceptibility to pregnancy could promote informed decision-making about contraception and reduce the risk of unintended pregnancy. Perceived severity relates to the perceived negative consequences of such pregnancies, such as health risks, economic burden, and potential impact on the well-being of both mothers and children. Perceived benefits encompass the advantages associated with contraception such as effective pregnancy prevention, birth spacing, and improved maternal and child health outcomes.

Perceived barriers involve obstacles or concerns individuals may have regarding contraceptive use, including cultural, religious, financial, or logistical factors. A study conducted in community contraception and sexual health clinics in Southeast Wales, which focused on understanding how health behavior models forecast the intention to use long-acting reversible contraception, discovered that anticipated obstacles significantly influenced the use of long-acting reversible contraception (8).

Cues to action refer to stimuli that prompt individuals to take action towards contraceptive use. These cues can include information from healthcare providers, educational campaigns, social networks, and personal experience. Self-efficacy represents an individual's confidence in their ability to adopt and maintain contraceptive behaviors, including the ability to navigate challenges and overcome barriers to contraception use (9).

Additionally, myriad modifying or enabling factors interact with an individual's views on pregnancy and decision-making, thereby influencing their use of contraception. This multifaceted aspect covers a broad array of factors, including demographic, social, structural, psychological, and reproductive elements, that have been found to predict patterns in contraceptive behavior (9). For example, certain groups tended to display a lower usage of highly effective contraceptive methods. Studies have shown that women living in rural areas and those with lower income levels are less inclined towards contraception than their counterparts residing in urban areas or those with a higher socioeconomic status (10). This disparity underscores the multifaceted nature of contraceptive behavior and the need for a comprehensive approach to improving access to and use of effective contraception.

Evidence suggests that the HBM serves as a suitable framework for exploring attitudes towards and use of postpartum contraception. Therefore, this study aimed to utilize the HBM as a guiding framework to examine the predictors of postpartum contraceptive use among Nigerian women. The HBM provides an interpretative lens for discussing the findings, thereby yielding insights that can directly inform evidence-based interventions.

## Materials and methods

### Study design and data collection

This study was a secondary analysis of cross-sectional data collected from the Demographic Health Survey conducted in Nigeria (NDHS) between August and September 2018 (11). This survey used computer-assisted personal interviews to collect information from the respondents. Two-stage cluster sampling was then performed. In the first stage, 1,400 enumeration areas (EAs) were selected with a probability proportional to the EA size. In the second stage, 30 households were selected from the clusters identified in the first stage, using equal probability systematic sampling. The process identified 41,688 households. The questionnaires were translated into three major languages (Igbo, Hausa, and Yoruba) and administered by trained fieldworkers.

Data for the current study included 28,041 women who had a live birth within 12 months prior to the survey and responded to the question on postpartum contraceptive use. The individual receiver of the 2018 dataset from the DHS was used because it contained all the study variables required. The outcome variable, “self-reported postpartum contraceptive use” was coded as “1” for respondents who reported having adopted contraceptive use and “0” for those who did not use contraceptives during the postpartum period. The selection of independent variables to predict postpartum contraceptive use was based on existing literature. The variables were categorized as follows: maternal age (15–24 years, 25–34 years, and 35–49 years), place of residence (urban or rural), region (Northwest, Northeast, Northcentral, Southeast, South-West, and South-South), religion (Catholic, Muslim, no religion), marital status (never married, married, and formerly married), educational level (no education, primary education, secondary education, or more), household wealth quintiles (poorest, poor, average, rich, and richest), knowledge of ovulatory cycle (yes or no), decision-maker for health care (wife, joint, husband, and someone else), and distance to health care facility (not a big problem or a big problem).

### Statistical analysis

Univariate and multivariate logistic regression analyses were conducted using STATA 13 software. Odds ratios (ORs) and 95% confidence intervals (CIs) were determined, and the level of significance was set at *p* < 0.05. The data were 98.5% complete, with only 1.5% missing data on healthcare decision-making variables. Because the data were missing at random, and only a small amount was missing, a complete case analysis was performed only on the rows of data that had no missing values. Multicollinearity was assessed using the variance inflation factor (VIF), which had a mean value of 1.2.

### Ethical consideration

The survey procedure and instruments used in the DHS were approved by the National Health Research Ethics Committee of the Federal Ministry of Health of Nigeria and the Ethics Committee of the Opinion Research Corporation Macro International Inc. (ORC Macro Inc., Calverton, MD, USA). The DHS program granted permission to use and access the Demographic Health Survey dataset.

## Results

### Descriptive statistics

Among 28,041 postpartum women studied, 27% used contraceptives (Table 1). Contraceptive use was highest in the 25–34 and 35–49 age groups with 28%–29% of women in both groups reporting contraceptive use. Usage was the lowest among the poorest quintiles (12%) and the highest among the richest quintiles (57%). In households where healthcare decisions were jointly made, contraceptive use was the highest (76%). Women who did not perceive distance from health care as a major problem reported lower contraceptive use (30%). Urban women showed a higher rate of contraceptive use (41%) than did rural women (19%). Women with tertiary education reported the highest use (65%), whereas those with no education reported the lowest use (13%). Christians showed an almost equal distribution of contraceptive use (48%), whereas usage among Muslim women was lower (19%). The highest rate of contraceptive use was observed in the Southeast region (54%), while the lowest rate was observed in the Northwest region (15%). Women who were married or in union had the highest use of contraceptives (43%).

**Table 1:** Frequencies of explanatory variables by postpartum contraceptive use.

Postpartum contraceptive use			
Variables	No	Yes	Total
Maternal age (years).			
15–24	3,654 (84%)	710 (16%)	4,364 (16%)
25–34	10,188 (71%)	4,229 (29%)	14,417 (51%)
35–49	6,622 (72%)	2,637 (28%)	9,260 (33%)
Total			**28,041 (100%)**
Knowledge of ovulatory cycle			
Yes	342 (5%)	7,235 (95%)	7,578 (5%)
No	1,230 (6%)	19,234 (94%)	20,464 (95%)
Total			**28,041 (100%)**
Wealth quintiles			
Poorest	6,217 (88%)	821 (12%)	7,038 (25%)
Poor	5,690 (84%)	1,097 (16%)	6,787 (24%)
Average	4,280 (72%)	1,695 (28%)	5,974 (21%)
Rich	2,842 (58%)	2,046 (42%)	4,887 (18%)
Richest	1,436 (43%)	1,919 (57%)	3,354 (12%)
Total			**28,041 (100%)**
Decision maker for healthcare			
Wife	1,581 (64%)	876 (36%)	2,457 (9%)
Joint decision	4,156 (24%)	12,890 (76%)	7,046 (26%)
Husband	14,149 (80%)	3,602 (20%)	17,751 (65%)
Someone else	49 (86%)	8 (14%)	57 (0.2%)
Total			**27,311 (100%)**
Distance to healthcare facility			
Not a big problem	13,930 (70%)	6,002 (30%)	19,932 (71%)
A big problem	1,575 (19%)	6,534 (81%)	8,110 (29%)
Total			**28,041 (100%)**
Settlement			
Rural	14,665 (81%)	3,530 (19%)	18,195 (65%)
Urban	5,799 (59%)	4,047 (41%)	9,846 (35%)
Total			**28,041 (100%)**
Region			
North central	2,368 (69%)	1,046 (31%)	3,414 (12%)
North east	4,094 (78%)	1,166 (22%)	5,260 (19%)
North west	10,689 (85%)	1,893 (15%)	12,582 (45%)
South east	1,085 (46%)	1,293 (54%)	2,378 (8%)
South south	1,047 (53%)	931 (47%)	1,978 (7%)
South west	1,182 (49%)	1,248 (51%)	2,430 (9%)
Total			**28,041 (100%)**
Mother's educational level			
No education	13,360 (87%)	1,924 (13%)	15,284 (55%)
Primary education	3,017 (66%)	1,554 (34%)	4,571 (16%)
Secondary education	3,572 (53%)	3,154 (47%)	6,726 (24%)
Tertiary education	515 (35%)	945 (65%)	1,460 (5%)
Total			**28,041 (100%)**
Religion			
No religion	171 (96%)	7 (4%)	178 (1%)
Christianity	4,118 (52%)	3,801 (48%)	7,919 (28%)
Islam	16,175 (81%)	3,770 (19%)	19,945 (71%)
Total			**28,041 (100%)**
Marital status of women			
Never married	205 (85%)	35 (15%)	240 (0.9%)
Married/in union	9,935 (57%)	7,375 (43%)	17,310 (97%)
Formerly married	324 (83%)	67 (17%)	391 (2%)
Total			**28,041 (100%)**

### Predictors of Contraceptive Use

All independent variables included were significantly associated with contraceptive use among postpartum women in Nigeria (Table 2). Women aged 25–34 years (AOR = 1.9; 95% CI: 1.7–2.2) and those 35–49 years (AOR = 2.3; 95% CI: 2.0–2.6) had significantly higher odds of postpartum contraceptive use compared to younger women aged 15–24 years. Knowledge of the ovulatory cycle was also associated with significantly higher odds of contraceptive use (AOR = 1.6; 95% CI: 1.4–1.8). Wealth status had a positive gradient, with the richest women having significantly higher odds of using contraceptives postpartum than those from the poorest households (AOR = 2.5; 95% CI: 2.0–2.9). The decision maker for healthcare within the household significantly impacted contraceptive use. Women who made joint decisions with their partners had a lower adjusted odds ratio (AOR = 0.8; 95% CI: 0.7–0.9) compared to those in households where the wife made the decisions. Women who did not perceive distance as a big problem had higher odds of using contraceptives (AOR = 1.2; 95% CI: 1.1–1.3) than women who did. Residence in urban areas was associated with higher odds of contraceptive use (AOR = 1.2; 95% CI: 1.1–1.3) than residence in rural areas. Educational level was positively associated with contraceptive use, with tertiary educated women having the highest odds (AOR = 3.3; 95% CI: 2.8–3.7), compared to women with no education. Regarding religion, compared to Christian women, Muslim women had lower odds of using contraceptives (AOR = 0.6; 95% CI: 0.5–0.6). In comparison to the North-Central region, the Northeast region had significantly lower odds of contraceptive use (AOR = 0.4; 95% CI: 0.3–0.5), while the North-West region had slightly lower odds (AOR = 0.7; 95% CI: 0.6–0.8). On the other hand, the South-East region had significantly higher odds of contraceptive use (AOR = 2.4; 95% CI: 2.1–2.6), as did the South-West region (AOR = 2.3; 95% CI: 2.0–2.6). Formerly married women (AOR = 2.2; 95% CI: 1.6–3.1) and married women (AOR = 2.6; 95% CI: 1.8–3.8) had higher odds of using contraceptives compared to those who were never married.

**Table 2:** Predictors of postpartum contraceptive use in the postpartum period.

Variables	OR, CI (95%)	*p*-value	OR, CI (95%)	*p*-value
Maternal age (years).				
15–24	1	–	1	–
25–34	2.1 (1.9–2.3)	<0.001	1.9 (1.7–2.2)	<0.001
35–49	2.1 (1.8–2.3)	<0.001	2.3 (2.0–2.6)	<0.001
Knowledge of ovulatory cycle				
No	1	–	1	–
Yes	1.7 (1.5–1.9)	<0.001	1.6 (1.4–1.8)	<0.001
Wealth quintiles				
Poorest	1	–	1	–
Poor	1.4 (1.3–1.6)	<0.001	1.2 (1.1–1.3)	<0.001
Average	2.9 (2.6–3.1)	<0.001	1.8 (1.6–1.9)	<0.001
Rich	4.7 (4.4–5.2)	<0.001	2.2 (2.0–2.5)	<0.001
Richest	8.0 (7.2–8.9)	<0.001	2.5 (2.0–2.9)	<0.001
Decision maker for healthcare				
Wife	1	–	1	–
Husband	0.4 (0.4–0.5)	<0.001	0.7 (0.6–0.8)	<0.001
Someone else	0.5 (0.3–0.9)	0.032	0.7 (0.4–1.5)	0.382
Joint decision	1.1 (0.9–1.2)	0.132	0.8 (0.7–0.9)	<0.001
Distance to healthcare facility				
A big problem	1	–	1	–
Not a big problem	1.7 (1.6–1.8)	<0.001	1.2 (1.1–1.3)	<0.001
Settlement				
Rural	1	–	1	–
Urban	2.4 (2.3–2.5)	<0.001	1.2 (1.1–1.3)	<0.001
Region				
North central	1	–	1	–
North east	0.4 (0.3–0.5)	<0.001	1.3 (1.2–1.5)	<0.001
North west	0.7 (0.6–0.8)	<0.001	0.7 (0.7–0.9)	<0.001
South east	2.4 (2.1–2.6)	<0.001	1.1 (1.0–1.3)	0.027
South west	2.3 (2.0–2.6)	<0.001	1.3 (1.1–1.5)	0.049
South south	1.5 (1.3–1.7)	<0.001	0.9 (0.8–1.0)	<0.001
Mother's educational level				
No education	1	–	1	–
Primary education	2.9 (2.7–3.2)	<0.001	1.7 (1.5–1.8)	<0.001
Secondary education	5.0 (4.6–5.3)	<0.001	2.2 (2.0–2.4)	<0.001
Tertiary education	10 (8.9–11.3)	<0.001	3.3 (2.8–3.7)	<0.001
Religion				
Christianity	1	–	1	–
Islam	0.3 (0.2–0.3)	<0.001	0.6 (0.5–0.6)	<0.001
No religion	0.04 (0.02–0.09)	<0.001	0.1 (0.04–0.2)	<0.001
Marital status of women				
Never married	1	–	1	–
Married/in union	2.2 (1.6–3.1)	<0.001	2.5 (1.8–3.5)	<0.001
Formerly married	2.6 (1.8–3.8)	<0.001	2.7 (1.8–3.9)	<0.001

## Discussion

This study aimed to analyze the predictors of postpartum contraceptive use in Nigeria using HBM. According to the findings of this study, the prevalence of postpartum contraceptive use among Nigerian women is 27%. Contraceptive use was higher among women who knew about their ovulation cycles, lived in urban areas, lived in the southeast, lived in the southwest, had no distance barriers to healthcare, and were 25–49 years old. Education, wealth, and marital status also increase the odds of contraceptive use. However, women who lived in the northeast and northwest regions or shared decision-making with their partners had lower odds.

Regional variations in postpartum contraceptive use within Nigeria can be ascribed to a complex interplay of socioeconomic, cultural, and religious dynamics, each of which serves as a significant modifying or enabling factor within the HBM framework. The northern regions of Nigeria, primarily Muslim-dominated, have cultural and religious beliefs that favor larger families. In these regions, it is believed that having more children is a way to pay homage to Allah, which significantly affects decisions regarding family size (12, 13). Consequently, the ideal number of children in these regions is 7.5, which is higher than the ideal number in the southern part of Nigeria, 5.3 (14) These cultural and ethnic orientations serve as significant modifying factors that influence perspectives on contraceptive use. In their qualitative study focusing on Somali women in Finland, Mohamed and Sundberg (15) demonstrated the influence of cultural beliefs on contraceptive attitudes; despite their relocation to Finland, Somali women continued to abide by cultural tenets. Northern regions also have high poverty rates (16) and limited healthcare services (17), which hinder the accessibility and availability of contraceptives and serve as enabling factors that further depress contraceptive use.

Conversely, the southern region demonstrated higher contraceptive use. In these regions, there is more health literacy and better healthcare access (18). These factors may contribute to a greater perception of contraceptive benefits and facilitate regular contraceptive use. However, in a review conducted by Kilfoyle et al. (19), no direct relationship was identified between health literacy status and actual contraceptive use. Nonetheless, other studies, including the present one, indicate that factors such as health insurance and lack of distance barriers to healthcare are integral to ensuring steady access to a wide range of contraceptive options (20–24).

Religion also plays a role, with Christian-majority southern regions generally demonstrating a more accepting attitude towards contraception compared to the Muslim-majority north (24). This acceptance can further decrease perceived barriers to contraceptive use. Religion is a key facet of the sociocultural fabric of Nigerian communities, granting religious leaders substantial influence in either deterring or encouraging effective adoption of contraception (25–27). An investigation into the impact of religious leaders on contraceptive use in Nigeria indicated significantly higher adoption of contraceptive methods among women exposed to family planning messages from their religious leaders than among those who did not (25). This underscores the potential role of religious leaders in positively shaping perceptions and attitudes toward contraception.

Age plays a substantial role in shaping contraceptive use in Nigeria. Women aged 25–49 are likely to have a higher perceived susceptibility to unwanted pregnancies, driving them to use contraceptives more than their younger counterparts aged 15–24. As demonstrated by Alo et al. (28) in 2020, the perceived severity of the implications of unplanned pregnancies may be more pronounced given their potentially closer proximity to achieving their desired family size. As women age, the perceived benefits of contraception, including family planning and birth spacing, are likely to become more evident. A study conducted in Ghana found that the majority of adolescent participants did not receive any significant information or reminders regarding contraceptive use (cues to action), and 70.7% did not perceive the information they received as important (29). Additionally, the majority of participants did not perceive adolescent pregnancy as a severe issue. However, a small majority recognized the benefits of contraceptive use, indicating some level of awareness regarding reproductive health choices.

Older women are often more educated and have a greater sense of autonomy over their health decisions than younger counterparts (30, 31). Heightened knowledge and empowerment can significantly influence perceived barriers and benefits of contraception. Several studies, including the present one, support this notion, demonstrating a positive correlation between education, control over health decisions, and contraceptive use (28, 32–34).

The perceived benefits of contraception such as controlled family planning and birth spacing may become more apparent with age. Simultaneously, perceived barriers may decrease, as these women may have more access to healthcare services and contraceptives. In Nigeria, contraceptive products are predominantly sourced from the private sector (35, 36). This makes contraception accessible to those who can afford it. Ezenwaka et al. (37) proposed that the high prevalence of teenage pregnancies in Nigeria signifies a lack of access to contraceptives among young women. Therefore, age is a pivotal enabling factor with the potential to affect accessibility, understanding, and use of contraceptive methods.

Consequently, the perceived lower financial barriers to contraceptive use likely explain the increasing trend of contraceptive use observed among the household wealth groups in this study as well as in previous research (18, 32, 35, 38). Highlighting the influence of financial constraints, and in contrast to the Ghanaian study (30), a study conducted in America (39) found that among low-income young women, perceived pregnancy risk did not significantly impact their use of contraception.

Furthermore, the increase in contraceptive use with age suggests more cues to action, likely because older women are more exposed to health information and services. This can be attributed to their age and potentially higher education levels, which provide opportunities for increased health literacy and access (19). Self-efficacy may also be higher among older women, possibly because of their greater knowledge, experience, and community or partner support, which enables them to confidently make contraceptive choices. Community and partner support are key factors in contraceptive use, particularly in Nigeria, where women still make contraceptive decisions based on their partners (18, 40).

Women knowledgeable about ovulation, residing in urban areas, and married also had higher postpartum contraceptive use. Understanding ovulation boosts perceived susceptibility to unplanned pregnancies, making contraception beneficial (4). Urban living often signifies fewer geographical and financial barriers to health care, thus facilitating contraceptive adoption. Consistent evidence, including that of the present study, supports urban residence as a determinant of contraceptive use (32, 33). Urban dwellers are also more likely to be educated, which increases their perceived severity of consequences and self-efficacy. Married women engaging in regular sexual activities may perceive contraception as critical for planned child spacing, thus driving its use. This aligns with previous studies in Nigeria, confirming that marital status is a significant predictor of contraceptive use (28).

### Research implications

These results highlight the importance of comprehensive reproductive health education, especially in relation to understanding menstrual cycles and ovulation. Educating women, particularly young and less-educated women, about these biological processes can increase their perceived susceptibility to unplanned pregnancies, increasing the likelihood of contraceptive use.

These findings also highlight the need for more targeted interventions in the northeastern and northwestern regions of Nigeria. Interventions in these regions should focus on addressing these barriers and adapting them to the unique sociocultural contexts. Engaging religious and community leaders in these regions to promote contraceptive use could be a particularly effective strategy given their influence on community norms and beliefs.

The results also suggest the importance of making contraceptive services more accessible and affordable, particularly for younger, less-educated, and lower-income women. Policies that expand the provision of contraceptive services in both public and private sectors could be crucial for increasing postpartum contraceptive use across different socioeconomic groups.

Additionally, this study underscores the role of women's autonomy in decision-making regarding contraceptive use. Therefore, interventions should also focus on empowering women, particularly in contexts where decision-making is predominantly controlled by their partners. This could involve education on reproductive rights, provide platforms for women to share their experiences, and challenge harmful norms that limit their decision-making autonomy.

### Strengths & limitations

Despite these potential limitations, this study's robustness was substantiated by some key strengths. First, a high response rate of approximately 99% minimizes non-response bias and enhances representativeness. Furthermore, the comprehensive sample size supported the generation of unbiased results.

One potential limitation is the self-reported nature of contraceptive use, which may introduce a reporting bias. However, the application of computer-assisted personal interviewing in this study provided a counterbalance by facilitating more thorough probing and more detailed information regarding contraceptive use. While it would have been insightful to incorporate explanatory variables, such as antenatal and postnatal care, these variables were not included because of substantial missing values. Nevertheless, the factors examined in this study provide valuable insights and contribute significantly to the literature on the factors influencing postpartum contraceptive use.

## Conclusion

In conclusion, this study identified the predictors of postpartum contraceptive use in Nigeria using the Health Belief Model. These findings emphasize the need for comprehensive, context-specific interventions that address socioeconomic, cultural, and structural barriers. Such interventions should focus on education, accessibility, empowerment, and engagement with religious and community leaders. Postpartum contraceptive use can be enhanced by adopting a holistic approach and reproductive health outcomes can be improved in Nigeria.

## Data Availability

The dataset analyzed for this study is from the DHS program. The 2018 dataset is publicly available to anyone. Requests to access the dataset should be directed to the DHS program at https://dhsprogram.com/.
